# Identification of quality markers for *Cyanotis arachnoidea* and analysis of its physiological mechanism based on chemical pattern recognition, network pharmacology, and experimental validation

**DOI:** 10.7717/peerj.15948

**Published:** 2023-09-11

**Authors:** Jingnan Hu, Yu Feng, Baolin Li, Fengxia Wang, Qi Qian, Wei Tian, Liying Niu, Xinguo Wang

**Affiliations:** 1 Hebei University of Chinese Medicine, Shijiazhuang, China; 2 Hebei Traditional Chinese Medicine Formula Granule Engineering & Technology Innovate Center, Shijiazhuang, China; 3 Quality Evaluation & Standardization Hebei Province Engineering Research Center of Traditional Chinese Medicine, Shijiazhuang, China

**Keywords:** *Cyanotis arachnoidea*, Quality markers, UHPLC, Chemical pattern recognition, Network pharmacology, Hypoglycemic, Ultra-high-performance liquid chromatography

## Abstract

*Cyanotis arachnoidea* C. B. Clarke is a traditional Chinese medicinal herb that has a limited clinical use in the treatment of diabetes mellitus (DM) in minority areas of Guizhou in China. However, few prior reports are available on the quality control of *Cyanotis arachnoidea*, and its quality markers and hypoglycemic mechanism are still unclear. The purpose of this study is to explore the quality markers (Q-markers) of *Cyanotis arachnoidea* and predict its hypoglycemic mechanism. In this study, ultra-high-performance liquid chromatography (UHPLC) fingerprint combined with chemical pattern recognition were performed, and four differential components were screened out as quality markers, including 20-Hydroxyecdysone, 3-*O*-acetyl-20-hydroxyecdysone, Ajugasterone C, and 2-*O*-acetyl-20-hydroxyecdysone. Network pharmacology analysis revealed 107 therapeutic target genes of *Cyanotis arachnoidea* in DM treatment, and the key targets were Akt1, TNF, IL-6, MAPK3, and JUN. The hypoglycemic mode of action of *Cyanotis arachnoidea* may be mediated by tumor necrosis factor (TNF) signaling, cancer, insulin resistance, and JAK-STAT pathways. Molecular docking analysis disclosed that the foregoing quality markers effectively bound their key target genes. An *in vitro* experiment conducted on pancreatic islet β-cells indicated that the forenamed active components of *Cyanotis arachnoidea* had hypoglycemic efficacy by promoting PI3K/Akt and inhibiting MAPK signaling. UHPLC also accurately quantified the quality markers. The identification and analysis of quality markers for *Cyanotis arachnoidea* is expected to provide references for the establishment of a quality control evaluation system and clarify the material basis and hypoglycemic mechanisms of this traditional Chinese medicine (TCM).

## Introduction

Diabetes mellitus (DM) is a complex and prevalent chronic metabolic condition. Its global incidence is increasing annually. DM is described as a disorder of glucose metabolism resulting from absolute or relative insulin insufficiency (Mustapha et al., 2021). According to the International Diabetes Federation, DM affects up to 463 million people in the age range of 20–79 years globally. China has approximately 114 million DM patients, and this number is expected to rise to 120 million by 2045 (Hu et al., 2019). DM is a disease associated with the most complications. The long-term increases in blood glucose associated with DM can cause damage to macro- and micro-vessels and adversely affect heart, brain, kidney, peripheral nerves, eyes, and feet (Xiaopeng et al., 2016). Consequently, extremely high disability and mortality rates are associated with DM, and it seriously threatens physical health and imposes a substantial socioeconomic burden on the patients afflicted with it.

Chinese herbal medicine has been administered to prevent and treat DM for thousands of years and has attracted research attention as it has multi-target and multi-pathway characteristics. The glycosides (Aswal et al., 2020), polyphenols (Riyaphan et al., 2021), phytosterones (Gu et al., 2021), flavones, and isoflavones (Xu et al., 2020) in Chinese herbal medicines provide efficacious glycemic control and ameliorate insulin resistance through various mechanisms. *Cyanotis arachnoidea* is a traditional Chinese herbal and ethnic medicine that is widely distributed in India, Sri Lanka, and the Yunnan Province of China. Ancient Chinese medicine texts reported that it has been used to treat nephritic edema, diabetes, rheumatoid arthritis, and other diseases (Lan & Su, 2017; Wang, 1975; Guizhou Institute of TCM, 1970; Bu, Zhang & Guo, 2013; Tan et al., 2002). Modern research has demonstrated that *Cyanotis arachnoidea* contains several phytosterones and is particularly rich in 20-Hydroxyecdysone (Nie & Qiu, 1987). This compound promotes *de novo* nucleic acid and protein synthesis (Omanakuttan et al., 2016), has pharmacological effects on glucose and lipid metabolism (Marschall et al., 2021), and has anti-inflammatory (Tang et al., 2020), antioxidant (Baev et al., 2022), neuroprotectant (Lim et al., 2020), and antitumor (Romaniuk-Drapała et al., 2021) activity. Previous studies (Chen, Xia & Qiu, 2006; Chen et al., 2017) showed that 20-Hydroxyecdysone ameliorated insulin resistance in HepG2 cells by activating the IRS-1/Akt pathway and upregulating GLUT4 and GLUT2 proteins. Hence, it has potential hypoglycemic efficacy. A clinical trial (Yi et al., 2018) demonstrated that a combination of *Cyanotis arachnoidea* and metformin significantly reduced the glycosylated hemoglobin and serum C-peptide levels in patients with type 2 diabetes mellitus (T2DM), thereby improving their typical symptoms. Nevertheless, active ingredients in *Cyanotis arachnoidea* and their hypoglycemic mechanisms remain unclear, and the adoption of this herb in clinical practice is severely limited. Furthermore, differences in the sources, harvest seasons, and processing methods of *Cyanotis arachnoidea* result in variability of its components, and effective quality control methods for *Cyanotis arachnoidea* are lacking.

Traditional Chinese medicine (TCM) herbs have complex compositions and multiple components, targets, and pathways. Thus, it is difficult to elucidate their modes of action. In 2016, quality markers (Q-markers) were proposed to evaluate and control TCM quality (Zhao et al., 2019; Bai et al., 2018). A Q-marker is a unique chemical component that is either inherent in TCM or forms when TCM herbs, decoctions, extracts, and patent medicines are prepared. It is closely related to the functional attributes of TCM, should be qualitatively and quantitatively determined, and could serve as a marker of TCM safety and efficacy (Liu, Guo & Liu, 2018). To search for Q-markers and hypoglycemic constituents in *Cyanotis arachnoidea*, and examine their hypoglycemic molecular mechanisms of action through the analysis of Q-markers and biomarkers, chemical pattern recognition and network pharmacology were performed to screen differential components, identify active ingredients and predict their potential targets. The TCM fingerprint is a comprehensive and quantifiable identification method that has been widely used for TCM quality control in combination with chemical pattern recognition techniques, such as hierarchical cluster analysis (HCA) and principal component analysis (PCA) (Jiang et al., 2022; Zhang et al., 2021). Network pharmacology is systematic and holistic and can be widely used to predict and research the multi-target mechanisms of TCM in the treatment of various diseases (Pan et al., 2022; Li et al., 2021; Wang, Cui & Li, 2019).

Molecular docking technology is a type of computer-aided drug design (CADD). This new drug research and development technology has rapidly developed to explore rational drug design. It uses information technology and exploits existing knowledge of related drugs and their targets to guide research in drug discovery and drug action mechanisms *via* calculations, simulations, and predictions (Baig et al., 2016). Digital CADD technology significantly shortens the time and lowers the cost of research. It decreases blindness, increases accuracy, and facilitates the discovery of high-activity, high-affinity drug molecules while targeting multiple sites. For these reasons, the advent of digital CADD technology is an important breakthrough in TCM development. Nevertheless, the models have low prediction accuracy, omit biologically active drug candidates, and do not consider component/drug toxicity (Kaur & Khatik, 2021). Molecular docking technology is important for further identifying the key targets of the active ingredients in TCMs by docking the ligands to their receptors and evaluating the stability of the docking system (Zhu et al., 2022).

**Figure 1:**
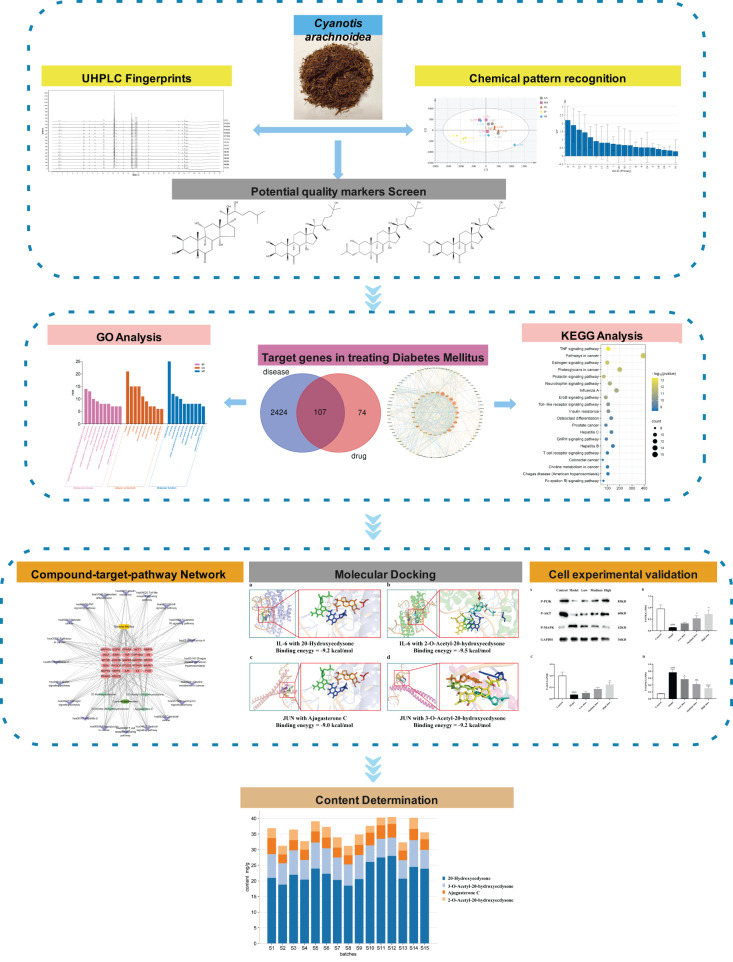
Detailed diagram of the overall study design. *: Compared with the model group, *P* < 0.05; **: Compared with the model group, *P* < 0.01; ***: Compared with the model group, *P* < 0.001; ****: Compared with the model group, *P* < 0.0001; ^####^: Compared with the blank control group, *P* < 0.0001.

In the present study, we used ultra-high-performance liquid chromatography (UHPLC) to establish a fingerprint reflecting the integrity of the chemical composition of *Cyanotis arachnoidea*. We screened potential quality markers and identified them by chemical pattern recognition technology. We used network pharmacology to predicte the hypoglycemic mechanisms of the components based on their quality markers and verified the latter by molecular docking and *in vitro* experiments. Finally, we quantitatively analyzed the quality markers by UHPLC. In conclusion, the present work devised a quality control evaluation method for *Cyanotis arachnoidea* and provided a reference for clarifying the pharmacodynamic mechanisms of the active ingredients of this TCM in DM. The experimental design is shown in Fig. 1.

## Materials and Methods

### Materials and reagents

#### Chinese medicinal materials

Fifteen batches of *Cyanotis arachnoidea* were purchased from China Shineway Pharmaceutical Group Ltd., Shijiazhuang, Hebei, China and authenticated by Professor Huixiang Yu of the Traditional Chinese Medicine Hospital of Chuxiong Yi Autonomous Prefecture, Yunnan, China. The dried roots were used in the experimental research, and the samples were deposited at the Hebei Traditional Chinese Medicine Formula Granule Engineering & Technology Innovate Center of the Hebei University of Chinese Medicine, Shijiazhuang, China.

#### Reference standards

20-Hydroxyecdysone (PU0362-0025MG), Ajugasterone C (PS0716-0010MG), 3-*O*-acetyl-20-hydroxyecdysone (PS210615-78-0005MG), and 2-*O*-acetyl-20-hydroxyecdysone (JOT-12130-0020MG) were obtained from Chengdu Push Biotechnology Co., Ltd. and Chengdu Pufei De Biotech Co., Ltd. (Sichuan, China). The purity of all the chemicals were greater than 98%. HPLC-grade methanol was obtained from Thermo Fisher Scientific (Waltham, MA, USA). Ultrapure water was generated by a Millipore Milli-Q Reagent Water System (EMD Millipore, Billerica, MA, USA). Chromatography-grade acetonitrile and formic acid were acquired from Sigma-Aldrich Corp. (St. Louis, MO, USA).

#### Cell lines

MIN6 islet β-cells (Lot No. CL-0674) were purchased from Procell Life Science & Technology Co., Ltd., (Wuhan, Hubei, China).

#### Reagents

The Roswell Park Memorial Institute (RPMI 1640) culture medium, the glucose assay kit and the radioimmunoprecipitation assay (RIPA) lysis solution were bought from Beijing Solarbio Science & Technology Co., Ltd., (Beijing, China). Protein bands were visualized with an enhanced chemiluminescence (ECL) detection kit (Thermo Fisher Scientific, Waltham, MA, USA). Anti-P-PI3K, anti-P-Akt, anti-P-MAPK and anti-GAPDH primary antibodies were acquired from bioWorld Technology Inc., (Dublin, OH, USA). Secondary antibodies were purchased from Zhongshanjinqiao Biotech (Beijing, China). Polyvinylidene fluoride (PVDF) transfer membranes were obtained from EMD Millipore.

### Preparation of the herbal extract and the reference substances

The herbs were pulverized and sifted, and the 15 *Cyanotis arachnoidea* sample batches are listed in Table S1. Herb powder (0.2 g) was dissolved in 25 mL of 70% (v/v) methanol in an Erlenmeyer flask and sonicated at constant ultrasound frequency (40 kHz; 250 W) for 45 min. Then 70% (v/v) methanol was added after the extraction to compensate for weight loss. The supernatant was removed, passed through a 0.22-μm filter membrane, and used in the subsequent analyses.

The reference substances were accurately weighed and dissolved in 10 mL chromatographic methanol to prepare stock solutions, and then were mixed at concentrations of 0.1723, 0.0251, 0.0539, and 0.0256 mg/mL. All solutions were stored at 4 °C until further use.

### UHPLC fingerprint analysis

#### UHPLC chromatography conditions

Chromatographic separation was performed in a Phenomenex Kinetex 2.6-μm C18 chromatographic column (100 mm × 3 mm; 2.6 μm) (Phenomenex, Torrance, CA, USA). The mobile phases were pure comprised pure acetonitrile (A) and 0.1% (v/v) formic acid in water (B), and the flow rate was 0.3 mL/min. The gradient elution program was as follows: 0–3 min, 5–13% A; 3–8 min, 13–15% A; 8–20 min, 15–35% A; 20–25 min, 35–95% A; 25–27 min, 95% A; 27–30 min, 95–5% B, and 30–33 min, 5% B. The column temperature was kept at 35 °C; the injection volume was 2.0 μL, and the detection wavelength was 248 nm.

#### Method validation

The same sample solution (S1) was measured sixfold in parallel to determine instrument precision. The relative standard deviation (RSD) values of the relative retention times and the peak areas under the common peaks were calculated using the peak of 20-Hydroxyecdysone as the reference. Repeatability was evaluated by analyzing nine independently prepared triplicate sample solutions at low (0.1 g), medium (0.2 g), and high (0.3 g) concentrations, each in triplicates. The stability of the method of analyzing the sample solutions was assessed at 0, 2, 4, 8, 12, and 24 h on the same day. Each sample solution was simultaneously assessed twice.

#### Fingerprint establishment and similarity analysis

Test solutions were prepared from powdered materials derived from the 15 sample batches and subjected to UHPLC analysis. The chromatograms generated were exported as CDF (computable document format) files and imported to the Similarity Evaluation System for Chromatographic Fingerprint of TCM (Version 2012) (Gu, Hall & Miles, 2016). The chromatogram for Batch 1 (S1) served as a reference and its common characteristic peaks were marked by multi-point correlation and automated Mark peak matching. A reference chromatogram (R) was generated by the median method. The degrees of similarity among the chromatograms for the 15 batches and that of R were calculated. Common peaks were identified by alignment with the reference compounds.

### Chemical pattern recognition analysis

#### HCA analysis

HCA was performed using the Ward method in SPSS 23.0 (IBM Corp., Armonk, NY, USA). The areas of the common peaks of the 15 batches were the variables and the Euclidean distance (Qin et al., 2022) was the metric.

#### PCA and PLS-DA analysis

PCA was performed in SIMCA v. 14.1 (https://umetrics-simca.software.informer.com/14.0/) to distinguish the quality of the materials from different regions and was based on the peak areas of the common peaks of the fifteen batches. Sample quality and discrepancy were judged according to the distances between sample points (Li et al., 2021). PLS-DA was conducted to screen out the differential components based on their variable importance in projection (VIP) values.

### Network pharmacology analysis

#### Prediction of target genes for the differential components and disease

Components contributing to the regional difference in material quality were identified by PLS-DA and used as candidate quality markers. The “SMILES” and “InChI” representations of the components were retrieved from the PubChem Compound database (https://www.ncbi.nlm.nih.gov/pubmed/). Standard 3D molecular structures were exported in structure-data file (SDF) format. Component targets were predicted using the BATMAN-TCM database (http://bionet.ncpsb.org.cn/batman-tcm/) (Liu et al., 2016) and based on their “InChI” representations (score > 20; *P* < 0.05). BATMAN-TCM is a bioinformatics analysis tool that elucidates the molecular mechanism of TCMs. It simultaneously analyzes multiple medicines and components and clarifies their mechanism at the molecular and systemic levels. Cytoscape v. 3.7.2 (https://cytoscape.org/download.html) was used to construct a component-target network.

Diabetes mellitus (DM)-associated target genes were collected from the GeneCards (Barshir et al., 2021) (https://www.genecards.org/), DrugBank (Wishart et al., 2018) (https://go.drugbank.com/), DisGeNET (Piñero et al., 2020) (https://www.disgenet.org/), and OMIM (Amberger et al., 2019) (https://omim.org/) databases by using the keyword/search term “diabetes mellitus”. The target genes were standardized by the UniProt database (UniProt Consortium, 2021) (https://www.uniprot.org/). The intersection target genes of component and DM were obtained on the Draw Venn Diagram platform (http://bioinformatics.psb.ugent.be/webtools/Venn/).

#### PPI network analysis and core target identification

A PPI network analysis of the intersecting target genes was constructed on the STRING database platform (Szklarczyk et al., 2021) (https://cn.string-db.org/). The confidence level for the interaction analysis in the network was >0.9. A topological analysis of the PPI network was performed using the “Analyze Network” function in Cytoscape v. 3.7.2, and the core targets were identified based on the degree, betweenness centrality (BC) and closeness centrality (CC) of each node.

#### GO and KEGG pathway enrichment analysis

GO annotation and KEGG pathway enrichment analyses were performed by importing the core targets to the DAVID v. 6.8 database (Jiao et al., 2021) (https://david.ncifcrf.gov). The biological processes and pathways potentially involved in the treatment of DM with *Cyanotis arachnoidea* were obtained at *P* ≤ 0.01 and Benjamini-Hochberg correction ≤0.01. Data for the active compounds and their core targets were imported into Cytoscape v. 3.7.2 to build a “drugs-active compounds-target genes-pathways” network.

### Molecular docking analysis

Molecular docking was performed on the four potential quality markers and the top five core targets according to their degree values. The crystal structures of the targets proteins were downloaded from the PDB database (Burley et al., 2022) (https://www.rcsb.org/) and saved in protein databank (PDB) file format. The PDB IDs of the top five target proteins (Akt1, TNF, IL-6, MAPK3, and JUN) were 4EKL, 2AZ5, 4NI9, 2ZOQ, and 5T01. Solvents and water molecules were removed with PyMOL software (https://www.pymol.org/). AutoDockTools v. 1.5.6 (https://autodocksuite.scripps.edu/adt/) was used to add hydrogens, calculate the atomic charges for the target proteins, and export the results in a pdbqt file format. Molecular docking was performed in AutoDock Vina (http://vina.scripps.edu/) after adjustment for the binding pocket radius size and the corresponding coordinate site to obtain reliable docking models for the core targets. Forty was the grid size of the docking box in blind docking, and the center X, Y, and Z axes of the top five key targets were (21.003, 2.523, 16.167), (−13.69, 71.605, 27.0), (−11.213, 33.474, 11.162), (15.007, −7.839, 24.613), and (−23.326, 17.31, 20.956), respectively.

### Experimental validation

Recovered MiN6 islet β-cells were inoculated into RPMI 1640 medium and incubated under a 5% CO_2_ atmosphere and at 37 °C. Cells in insulin solution with the concentration of 1 × 10^−7^ mol/L comprised the model group. Cells in buffer solution (no insulin) comprised the control group. The cells in both groups were subjected to 0.1 mL of 10 mM glucose solution. After continued culture for 24 h, the glucose concentration in the supernatant of the model group was significantly higher than in that of the control group. Hence, the cellular insulin resistance model was successfully established. The cells were then collected and their density was adjusted to 1 × 106/L. Then 0.1 mL cell suspension was added to a 96-well culture plate. Chinese herbal extract (64 mg) was weighed out and dissolve in 10 mL of serum-free medium to obtain a 6,400 mg/L storage solution. The latter was diluted to 100, 200, and 400 mg/L for administration to the low, medium and high dose groups, respectively. Each group had triplicate wells. 0.1 mL of 10 mmol/L glucose was simultaneously added to all cells in each group. The cells were then incubated for 24 h, and the glucose concentrations in the cell culture media were measured by the glucose oxidase method (Xuguang et al., 2019).

The expression levels of the phosphorylated PI3K (p-PI3K), Akt (p-Akt), and MAPK (p-MAPK) proteins were detected by western blot. After 24 h cell culture, the supernatants were removed, 200 μL cell lysate was added, and the cells were incubated at 4 °C overnight and centrifuged at 4 °C and 12,000 rpm for 10 min. The UV method and 10% sodium dodecyl sulfate-polyacrylamide gel electrophoresis (SDS-PAGE) were used to determine the protein content in the supernatants. to determine the protein content by UV method, and carried out 10% SDS-PAGE electrophoresis. The polyvinylidene fluoride (PVDF) membrane was then subject to a constant 250 mA for 60 min, and blocked with skim milk at 37 °C for 2 h. The membrane was then washed with phosphate-buffered saline (PBS), and a mixture of PBS-diluted primary antibodies (anti-p-PI3K, 1:3,000; anti-p-MAPK, 1:1,500; anti-p-Akt, 1:1,200; and anti-GADPH, 1:2,000) was added. The membrane was incubated overnight at 4 °C, washed again, and subjected to horseradish peroxidase (HRP)-labeled secondary antibody. Then the enhanced chemiluminescence (ECL) method was used to expose and develop the membrane with a gel imager. Finally, GAPDH was used as the internal reference and the protein bands were scanned and analyzed in Tanon Gis software to determine their grayscale values. The relative protein expression level equals the grayscale values of the target protein bands divided by those of the GAPDH protein bands.

### Content determination of the potential quality markers

#### Method validation

The mixed reference solutions were serially diluted to obtain gradient samples at six different concentrations (*n* = 6). The mixed reference solutions (0.2, 0.5, 1, 2, 3, and 5 μL) were placed in 10-mL volumetric flasks, diluted to the mark with 70% (v/v) methanol, and shaken. In this manner, a concentration series of mixed reference solutions for the four components were obtained. Two microliters of the reserve solution were subjected to UHPLC analysis as previously described. The reference substance concentrations were plotted on the abscissa (x-axis; X) while the peak areas were plotted on the ordinate (y-axis; Y). Standard curves were then drawn and the regression equations and linear ranges were calculated for each component.

An appropriate amount of the mixed reference solution was diluted with an equal volume of 70% (v/v) methanol. Then 2 μL diluted mixed reference solution was accurately pipetted and injected into the UHPLC. The chromatographic analysis was performed in duplicate. The precision, repeatability and stability of the determination method in methodological validation was refered to the UHPLC fingerprint. The peak area of each compound was recorded and the results of the RSD were calculated to determine sample precision.

#### Quantitative analysis

The 15 sample batches were accurately weighed and prepared as test solutions. UHPLC was then performed to establish the peak areas. The contents of the four potential quality markers were calculated and determined by measuring the peak areas.

### Statistical analysis

All data were expressed as ( $\bar{x}$ ± s) and the statistical analyses were performed in GraphPad Prism v. 9.4.0 (GraphPad Software, La Jolla, CA, USA). One-way ANOVA was used to compare data among different groups. Multiple comparisons were made using Tukey’s test. *P* < 0.05 was considered statistically significant.

## Results and discussion

### Establishment of UHPLC fingerprints

**Figure 2:**
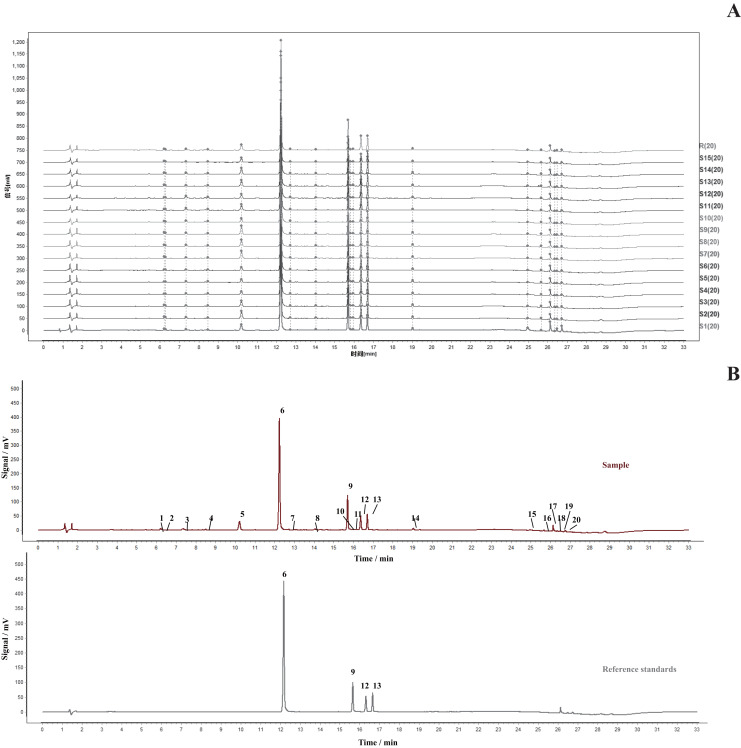
Identification and fingerprints of 15 sample batches of *Cyanotis arachnoidea*. UHPLC fingerprints of 15 *Cyanotis arachnoidea* samples batches (S1–S15) and reference fingerprint (S1). Twenty common characteristic peaks were selected (A). Comparison of the UHPLC chromatograms for the samples and reference standards identified four components. Peaks 6, 9, 12, and 13 correspond to 20-Hydroxyecdysone, 3-*O*-Acetyl-20-hydroxyecdysone, Ajugasterone C, and 2-*O*-Acetyl-20-hydroxyecdysone, respectively (B).

The fifteen *Cyanotis arachnoidea* samples batches were collected in five different regions of Yunnan Province including Anning (S1–S3), Menghai (S4–S6), Jinning (S7–S9), Jinping (S10–S12), and Jinghong (S13–S15), and their fingerprints were established by UHPLC. Twenty common characteristic peaks were automatically selected as the fingerprints. Peak-6 served as the reference for a standard R chromatogram that was determined by the median method and superimposed on the fingerprints (Fig. 2A). Comparison of the UHPLC chromatograms and the peak retention times of the samples and the mixed control solution, four components were identified, including 20-Hydroxyecdysone (peak-6), 3-*O*-Acetyl-20-hydroxyecdysone (peak-9), Ajugasterone C (peak-12), and 2-*O*-Acetyl-20-hydroxyecdysone (peak-13) (Fig. 2B).

According to the Similarity Evaluation System for Chromatographic Fingerprint of TCMs (Version 2012), the similarity values of the S1–S15 samples were in the range of 0.996–1.000 and generally remained at high levels (Table S1). Hence, the overall quality of 15 batches of *Cyanotis arachnoidea* was relatively stable, but the content of each component might be different. For this reason medicinal materials from different sources might vary in terms of their quality stability and homogeneity. Systematic studies of the chemical components of TCM indicate that their fingerprints could be used in their analysis and the determination of their overall quality control.

The precision, repeatability, and stability of the foregoing method were validated, and the RSDs of the average retention time and the peak area were used to evaluate it. According to the precision, repeatability, and stability tests, the RSD values for the average retention times and peak areas were all <0.2% and <2.89%, <0.14% and <2.81%, and <0.28% and <2.96%, respectively (Table S2). Validation of the method demonstrated good instrument precision, excellent repeatability, and sample solutions that were stable for 24 h. Therefore, the method could be used in the qualitative identification of *Cyanotis arachnoidea*.

### Chemical pattern recognition analysis

#### Hierarchical cluster analysis

**Figure 3:**
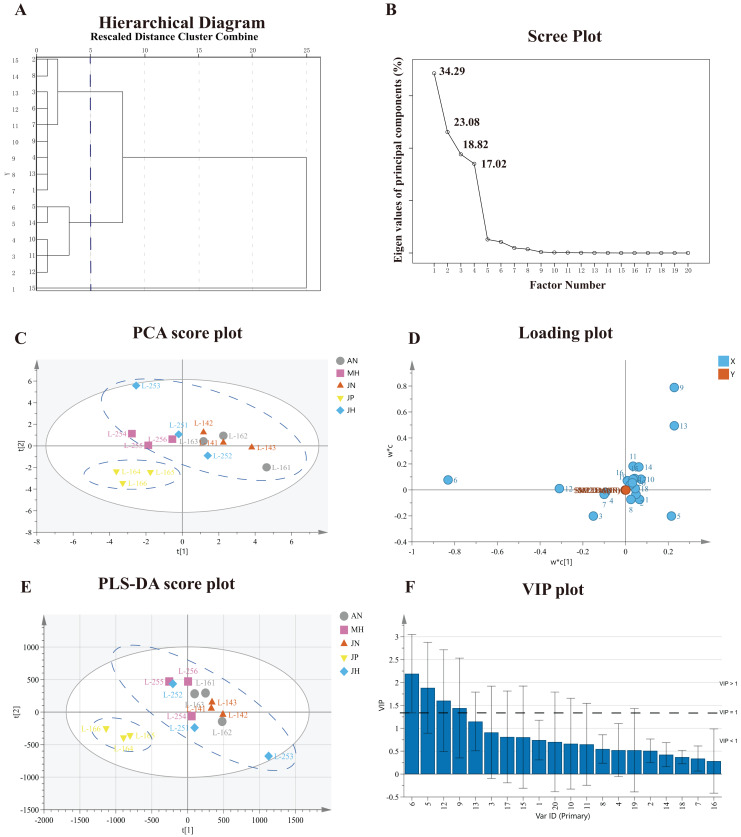
Screening of potential quality markers *via* chemical pattern recognition techniques. (A) Hierarchical clustering analysis (HCA) of fifteen *Cyanotis arachnoidea* sample batches. (B) Scree plot of *Cyanotis arachnoidea* samples. Curve steepness increases with the component contribution rate. (C) PCA score plot of *Cyanotis arachnoidea* samples. (D) Loading plot of *Cyanotis arachnoidea* samples. Variable influence increases with the distance from the origin. (E) PLS-DA score plot of *Cyanotis arachnoidea* samples and cluster separation. (F) Importance variable in projection (VIP) scores of the peak area variables of the samples in the PLS-DA model.

The twenty common characteristic peaks were subjected to HCA in SPSS v. 23.0 to validate the results of the similarity assessment. The average distance between groups served as the measurement standard. At a distance of five, 15 sample batches were divided into two main clusters (Fig. 3A). S1–S4, S6–S9, and S13 were assigned to Cluster І while all others were assigned to Cluster ІІ. After standardized processing and operation of the original data matrix, the four main components with eigenvalues greater than 1 were extracted, and their cumulative contribution rate has reached to be 93.21%. The components and their corresponding eigenvalues were shown in a scree plot (Fig. 3B). Curve steepness increases with the components contribution rate. In accordance with similarity analysis, these results indicated that the chemical constituents in *Cyanotis arachnoidea* samples were different although there were high similarities among the samples collected in various areas.

#### Principal component analysis (PCA) and partial least squares discriminant analysis (PLS-DA)

Principal component analyses (PCA) and partial least squares discriminant analyses (PLS-DA) were conducted in Simca-P v. 14.1 to screen components as potential quality markers and identify the differences in the chemical constituents and quality of the *Cyanotis arachnoidea* samples. The areas of the twenty common characteristic peaks generated by the fifteen sample batches were used as the original variables.

The clusters containing similar samples and the separation of the sample classes can be displayed in score plost (Yu et al., 2022). The PCA score plot (Fig. 3C) showed that the 15 batches of samples were divided into two clusters. The S10–S13 samples from Jinping were in Cluster І, all others were in Cluster ІІ, and the results were roughly consistent with those of the HCA. The influences of the variables on the principal component weight increased with the distance from the origin. The eigenvalues of the four principal components extracted were 8.82, 3.17, 1.49, and 1.34, respectively. The cumulative variance contribution rate for the first four principal components have reached was 98.9%, indicating that the four principal components can reflect the basic characteristics and most of the information for *Cyanotis arachnoidea*. Loading plots showed that the similarities between the variables and their effects on the scores. Loading and influence on the scores increase with the distance of the variables from the origin (Pan et al., 2022). Thus, the chromatographic peaks 3, 5, 6, 9, 12 and 13 contributioned the most to the principal components (Fig. 3D). These six components might mainly account for the observed differences among samples of different origins.

PLS-DA was also performed to demonstrate the differences in *Cyanotis arachnoidea* of different origins. The PLS-DA also divided the 15 sample batches into two clusters (Fig. 3E). Cross-validation disclosed that the model interpretation rate parameter R2X was 0.932, and the prediction ability parameters Q2 was 0.574, indicated that the model was both stable, reliable, and with high predictive abilities. Then the variable importance in projection (VIP) values of the 20 sample peak area variables in the PLS-DA model were extracted to screen the components explaining the difference among the fifteen sample batches. The components 20-Hydroxyecdysone, 3-*O*-acetyl-20-hydroxyecdysone, Ajugasterone C and 2-*O*-acetyl-20-hydroxyecdysone all had VIP scores > 1 (Jiang et al., 2022). For this reason, they were deemed potential quality markers distinguishing *Cyanotis arachnoidea* samples from different origins (Fig. 3F).

### Network pharmacology analysis

#### Target gene screening and interaction network construction

**Figure 4:**
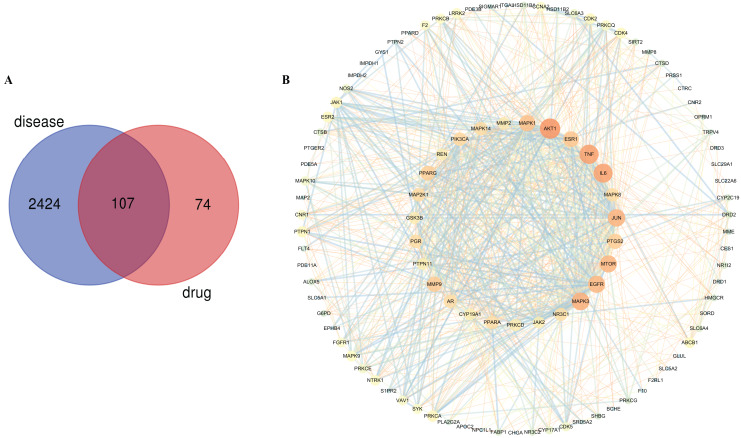
Target genes predcition of *Cyanotis arachnoidea* in DM treatment. After intersection of 181 compounds target genes and 2,571 disease target genes, 107 target genes associated with DM treatment by *Cyanotis arachnoidea* were obtained (A). The PPI network of the intersecting target genes was established and 27 core target genes were selected according to their degree value (B). The nodes in the inner circle represented the core target genes and the node size and color represented the degree. The node from tiny to large is represented by the degree from low to high, and the color is orange to cyan. Edge betweenness was expressed by edge size and color. The border between low and high symbolized the transition from thin to thick and the change in hue from orange to cyan. The darker the color, the more important of the targets in DM treatment.

A network pharmacological method was performed to predict the pharmacodynamic activity of the quality markers, explore the underlying hypoglycemic mechanisms of *Cyanotis arachnoidea*, and identify the potential targets and corresponding pathways of *Cyanotis arachnoidea* in the treatment of DM. Chemical information for four potential quality markers was obtained from the PubChem database and 181 predictive targets were obtained from BATMAN-TCM. DM-related targets were identified by searching the DisGeNET, GeneCards, DrugBank, and OMIM databases using the keyword “diabetes mellitus”. We obtained 2,571 genes related to DM deleting duplicated targets. The *Cyanotis arachnoidea* and DM targets were uploaded to the online tool Venny v. 2.8 (http://bioinfogp.cnb.csic.es/tools/venny/) for mapping and intersection. A total of 107 intersection target genes were obtained and they were considered to be the potential targets of *Cyanotis arachnoidea* in DM treatment (Fig. 4A).

The intersecting target genes were imported into the STRING database for the construction of a protein-protein interaction (PPI) network. There were 107 nodes (target proteins) and 878 edges (interactions) in the network (Fig. 4B). Cytoscape v. 3.7.2 software was used to visualize the protein interactions and the topological analysis was performed by the NetworkAnalyzer plugin. Based on the degree value, the betweenness centrality (BC) and the closeness centrality (CC) of each node in the network, take more than double median as the standard, 27 target genes were selected with degree ≥ 24, BC ≥ 0.0056 and CC ≥ 0.9722. These were the core targets implicated in DM treatment by *Cyanotis arachnoidea* (Table 1).

**Table 1:** Detailed diagram of the overall study design.

No.	Name	Betweenness centrality	Closeness centrality	Degree
1	AKT1	0.1443	0.7047	61
2	TNF	0.0696	0.6731	56
3	IL6	0.0712	0.6731	55
4	MAPK3	0.0390	0.6442	50
5	JUN	0.0296	0.6364	48
6	EGFR	0.0574	0.6325	47
7	MTOR	0.0597	0.6325	47
8	MAPK1	0.0183	0.6105	43
9	ESR1	0.0288	0.6034	41
10	MMP9	0.0379	0.5966	39
11	PTGS2	0.0308	0.5966	38
12	PPARG	0.0343	0.5866	37
13	PIK3CA	0.0091	0.5526	32
14	MAPK8	0.0076	0.5738	31
15	MAPK14	0.0153	0.5645	31
16	MAP2K1	0.0058	0.5615	31
17	PGR	0.0211	0.5556	31
18	AR	0.0233	0.5615	31
19	PPARA	0.0348	0.5707	31
20	NR3C1	0.0202	0.5585	30
21	MMP2	0.0069	0.5469	26
22	PRKCD	0.0176	0.5224	26
23	REN	0.0319	0.5497	25
24	GSK3B	0.0093	0.5440	25
25	PTPN11	0.0032	0.5198	24
26	CYP19A1	0.0129	0.5385	24
27	JAK2	0.0085	0.5303	24

#### Pathway analysis and construction of the compound-target-pathway network

**Figure 5:**
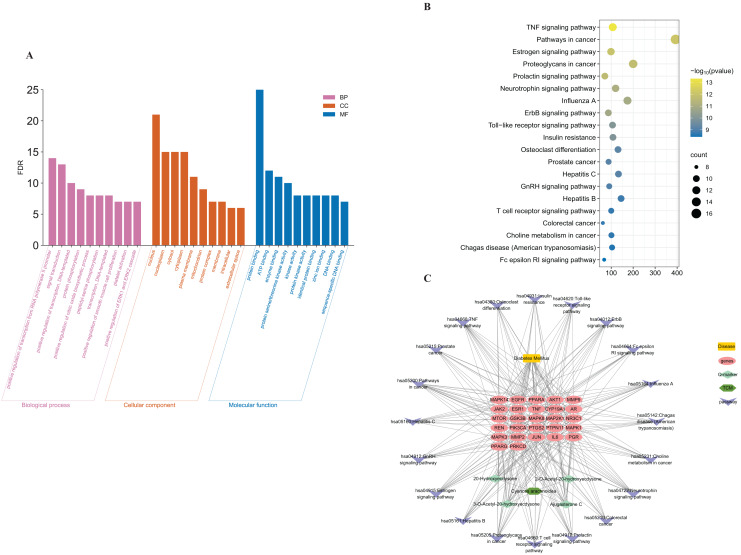
Enrichment of GO and KEGG pathway analysis and compound-target-pathway network establishment. GO enrichment analysis including the top 10 BP, MF, and CC entries. Histogram height is proportional to the number of biological processes expressed by the enrichment score (A). In the KEGG pathway analysis (top 20), the node size represents the number of enriched target genes, and the node color progression from green to red indicates a decrease in *P* value (B). The compound**-**target**-**pathway network was constructed by linking the components, and their potential targets, and biological pathways. The yellow, red, dark green, light green, and purple nodes represent the disease, core target genes, herb, four quality markers, and pathways, respectively (C).

In order to predict the key pathways of *Cyanotis arachnoidea* for DM treatment, GO analysis and KEGG pathway enrichment analyses of 27 core targets were conducted using the DAVID database. For the GO analysis, a total of 295 GO entries with *P* < 0.05 were obtained, including 230 biological process (BP), 42 cell component (CC), and 23 molecular function (MF). The top 10 BP, CC, and MF entries were selected for visualization (Fig. 5A). The GO analysis of the core target genes showed that the “biological” process term included mainly RNA polymerase II promoter transcription, protein phosphorylation, protein kinase activity, protein serine/threonine kinase activity, biological membrane protein interaction, and sequence-specific DNA binding.

A total of 101 KEGG pathways were obtained from the DAVID database and the top 20 were selected based on their-log *P* values (Fig. 5B, Table 2). The KEGG pathway enrichment analysis showed that the 27 core target genes were related mainly to the tumor necrosis factor (TNF), estrogen signaling, insulin resistance, JAK-STAT, and other pathways. Hence, *Cyanotis arachnoidea* may treat DM by acting on multiple signaling pathways. Moreover, there may be complex interactions among these pathways.

**Table 2:** The KEGG enrichment analysis information.

ID	Term	Count	*P* value	Genes
hsa04668	TNF signaling pathway	12	4.87E−14	IL6/MAP2K1/JUN/MAPK8/PIK3CA/MAPK1/AKT1/MAPK14/PTGS2/TNF/MMP9/MAPK3
hsa05200	Pathways in cancer	16	7.53E−13	GSK3B/MAP2K1/JUN/MMP2/PTGS2/MMP9/EGFR/MTOR/AR/IL6/MAPK8/PIK3CA/AKT1/MAPK1/PPARG/MAPK3
hsa04915	Estrogen signaling pathway	11	1.05E−12	MAP2K1/JUN/PIK3CA/MMP2/PRKCD/MAPK1/AKT1/ESR1/MMP9/EGFR/MAPK3
hsa05205	Proteoglycans in cancer	13	1.78E−12	MAP2K1/MMP2/PTPN11/MAPK14/ESR1/TNF/MMP9/EGFR/MTOR/PIK3CA/AKT1/MAPK1/MAPK3
hsa04917	Prolactin signaling pathway	10	2.14E−12	GSK3B/MAP2K1/MAPK8/PIK3CA/MAPK1/AKT1/JAK2/MAPK14/ESR1/MAPK3
hsa04722	Neurotrophin signaling pathway	11	7.51E−12	GSK3B/MAP2K1/JUN/MAPK8/PIK3CA/PRKCD/MAPK1/AKT1/PTPN11/MAPK14/MAPK3
hsa05164	Influenza A	12	1.10E−11	GSK3B/IL6/MAP2K1/JUN/MAPK8/PIK3CA/MAPK1/AKT1/JAK2/MAPK14/TNF/MAPK3
hsa04012	ErbB signaling pathway	10	1.42E−11	GSK3B/MAP2K1/JUN/MAPK8/PIK3CA/MAPK1/AKT1/EGFR/MTOR/MAPK3
hsa04620	Toll-like receptor signaling pathway	10	8.73E−11	IL6/MAP2K1/JUN/MAPK8/PIK3CA/MAPK1/AKT1/MAPK14/TNF/MAPK3
hsa04931	Insulin resistance	10	1.04E−10	GSK3B/IL6/MAPK8/PIK3CA/PRKCD/AKT1/PTPN11/PPARA/TNF/MTOR
hsa04380	Osteoclast differentiation	10	5.94E−10	MAP2K1/JUN/MAPK8/PIK3CA/MAPK1/AKT1/PPARG/MAPK14/TNF/MAPK3
hsa05215	Prostate cancer	9	6.72E−10	GSK3B/AR/MAP2K1/PIK3CA/MAPK1/AKT1/EGFR/MTOR/MAPK3
hsa05160	Hepatitis C	10	6.81E−10	GSK3B/MAPK8/PIK3CA/MAPK1/AKT1/MAPK14/PPARA/TNF/EGFR/MAPK3
hsa04912	GnRH signaling pathway	9	8.83E−10	MAP2K1/JUN/MAPK8/MMP2/PRKCD/MAPK1/MAPK14/EGFR/MAPK3
hsa05161	Hepatitis B	10	1.48E−09	IL6/MAP2K1/JUN/MAPK8/PIK3CA/MAPK1/AKT1/TNF/MMP9/MAPK3
hsa04660	T cell receptor signaling pathway	9	1.89E−09	GSK3B/MAP2K1/JUN/PIK3CA/MAPK1/AKT1/MAPK14/TNF/MAPK3
hsa05210	Colorectal cancer	8	1.96E−09	GSK3B/MAP2K1/JUN/MAPK8/PIK3CA/MAPK1/AKT1/MAPK3
hsa05231	Choline metabolism in cancer	9	2.05E−09	MAP2K1/JUN/MAPK8/PIK3CA/MAPK1/AKT1/EGFR/MTOR/MAPK3
hsa05142	Chagas disease (American trypanosomiasis)	9	2.59E−09	IL6/JUN/MAPK8/PIK3CA/MAPK1/AKT1/MAPK14/TNF/MAPK3
hsa04664	Fc epsilon RI signaling pathway	8	3.81E−09	MAP2K1/MAPK8/PIK3CA/MAPK1/AKT1/MAPK14/TNF/MAPK3

#### Compound-target-pathway network construction

The compound-target-pathway network contained four potential quality markers, 27 core targets, and 20 pathways, and was constructed with Cytoscape v. 3.7.2 (Fig. 5C). The network contained 282 edges and 53 nodes representing 27 core target genes, four active components, one disease, one compound prescription, and the top 20 KEGG pathways. The results indicated that the potential quality markers in *Cyanotis arachnoidea* might play roles in the treatment of DM by regulating multiple target proteins and acting on multiple pathways.

The compound-target-pathway network revealed that all four quality markers were phytosterones. According to modern pharmacology, 20-Hydroxyecdysone has antioxidant, anti-microbial, anti-inflammatory, and glucose-regulating activity. DM is closely associated with oxidative stress (Lima, Moreira & Sakamoto-Hojo, 2021), inflammation (Takahashi et al., 2022) and cancer (Carstensen et al., 2016). The disorders of glucose and lipid metabolism associated with DM activate the immune system, and macrophages synthesize and secrete various proinflammatory factors such as TNF-α, IL-1β, and IL-6 (Oliver et al., 2010). These substances affect the blood and the paracrine, the sensitivity of target tissues to insulin, and the function of islet β-cells (Kitade et al., 2012). Analyses of the KEGG enrichment and the core target genes revealed that the hypoglycemic efficacy of *Cyanotis arachnoidea* may be attributed to the amelioration of insulin resistance, antioxidant and anti-inflammatory activity, and impact on the TNF-α signaling pathway.

### Molecular docking verification

**Figure 6:**
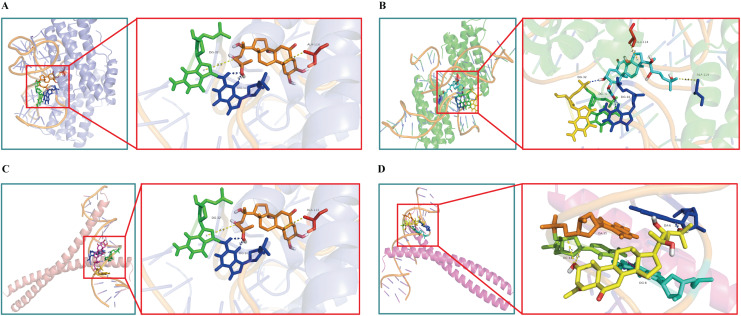
Molecular docking diagrams. (A) 20-Hydroxyecdysone with IL-6 (Binding eneygy = −9.2 kcal/mol); (B) 2-*O*-Acetyl-20-hydroxyecdysone with IL-6 (Binding eneygy = −9.5 kcal/mol); (C) Ajugasterone C with JUN (Binding eneygy = −9.0 kcal/mol); (D) 3-*O*-Acetyl-20-hydroxyecdysone with JUN (Binding eneygy = −9.2 kcal/mol). Proteins shown in cartoon mode are represented by different colors. Hydrogen bonds are depicted as yellow dotted lines, and the ligands are represented by sticks.

Molecular docking strategy was used to further verify the activity of the quality markers and disclose the binding pattern between the active components and the core genes. AutoDock Vina was used to dock the four potential quality markers with the top five target proteins. The latter were selected according to their degree values and included Akt1 (PDB ID: 4EKL), TNF (PDB ID: 2AZ5), IL-6 (PDB ID: 4NI9), MAPK3 (PDB ID: 2ZOQ), and JUN (PDB ID: 5T01). The binding energy docking results were listed in Table 3. Docking groups with the strongest target binding energies were selected for each component and visualized in PyMOL (Fig. 6). The results show that all the potential quality markers had molecular binding energies less than −6.7 kcal/mol with their corresponding core targets, indicating that each compound had a good affinity for its target proteins. The four quality markers could be the major active ingredients of *Cyanotis arachnoidea* for DM treatment. Moreover, their antihyperglycemic effect may be mediated by regulating the activity of Akt1, TNF, IL-6, MAPK3, and JUN activity.

**Table 3:** Binding energies of the quality markers with top five core targets.

Number	Compound	Binding energies (kcal/mol)				
		AKT1	TNF	IL-6	MAPK3	JUN
1	20-Hydroxyecdysone	−8.2	−8.1	−9.2	−8.6	−9.1
2	3-*O*-Acetyl-20-hydroxyecdysone	−7.7	−7.2	−9.1	−8.3	−9.2
3	Ajugasterone C	−7.7	−8.5	−8.6	−8.3	−9.0
4	2-*O*-Acetyl-20-hydroxyecdysone	−8.2	−7.5	−9.5	−8.7	−9.5

### *In vitro* validation

**Figure 7:**
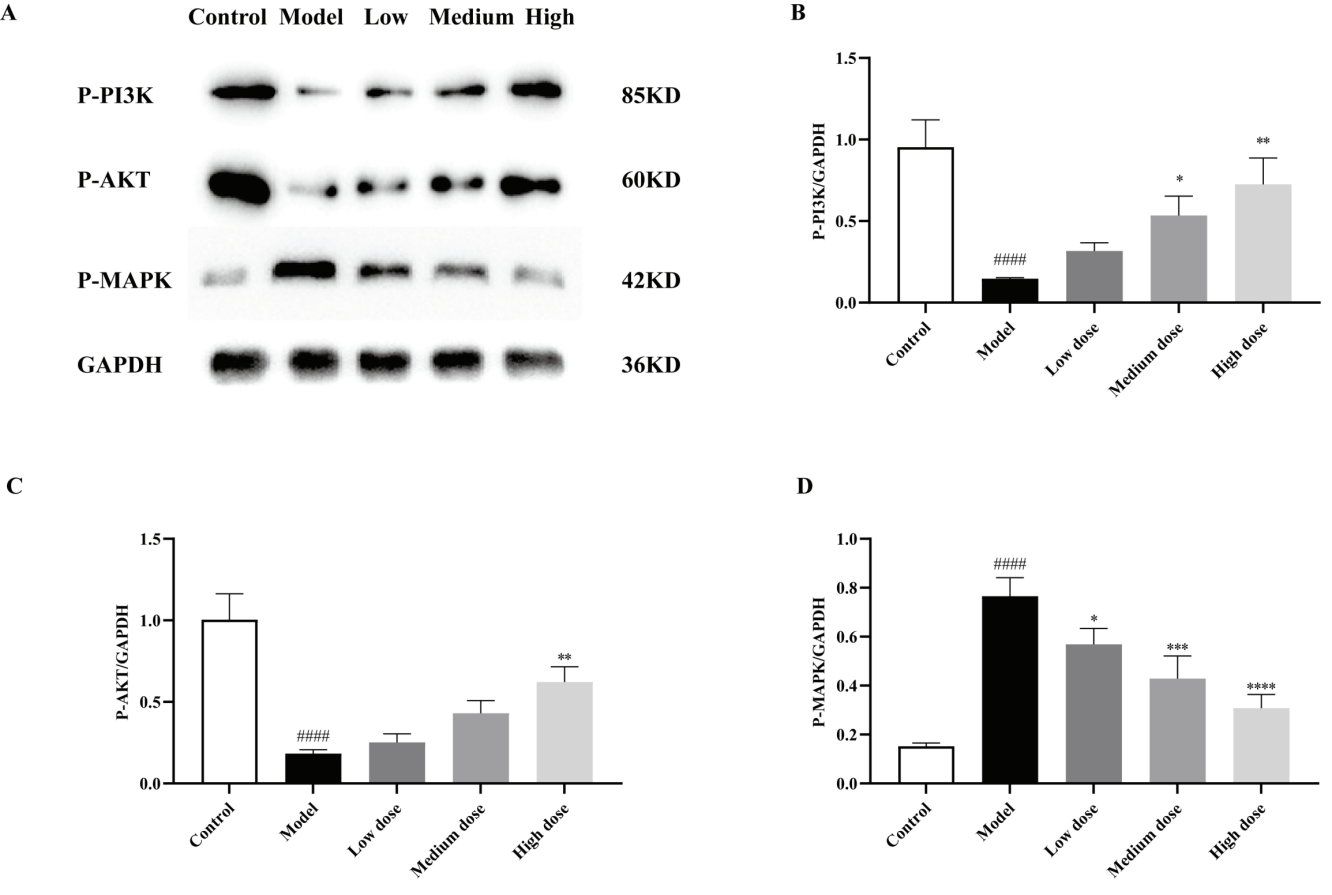
Protein expression results (±*s*, *n* = 3). (A) Electrophoregram of the p-PI3K, p-Akt and p-MAPK proteins in the control, insulin resistance model, and various *Cyanotis arachnoidea* extract intervention groups. (B) Relative phosphorylated PI3K protein expression. (C) Relative phosphorylated Akt protein expression. (D) Relative phosphorylated MAPK protein expression. *: Compared with the model group, *P* < 0.05; **: Compared with the model group, *P* < 0.01; ***: Compared with the model group, *P* < 0.001; ****: Compared with the model group, *P* < 0.0001; ^####^: Compared with the blank control group, *P* < 0.0001.

It can be seen from Table S3 that the cell supernatant glucose concentration was significantly higher for insulin resistance than the control group (*P* < 0.05). It shows that the *Cyanotis arachnoidea* extract increased the glucose uptake and utilization by the islet β-cells, effectively lowered glucose concentration, alleviated insulin resistance, and exhibited hypoglycemic activity.

p-PI3K/GAPDH and p-Akt/GAPDH were 0.15 ± 0.01 and 0.18 ± 0.02 in insulin resistance group, and were significantly lower than those in the blank control group (*P* < 0.05). p-MAPK/GAPDH was 0.77 ± 0.06 which was significantly higher than that of the blank control group (*P* < 0.05). Compared with the insulin resistance group, the p-PI3K and p-Akt protein expression levels significantly increased while that of p-MAPK significantly decreased with increasing *Cyanotis arachnoidea* extract dose (*P* < 0.05; Figs. 7A–7B and Table S4). The PI3K/Akt signaling pathway plays an important role in blood glucose regulation. Insulin resistance is related to the dysregulated signal transduction of the PI3K/Akt pathway and the overexpression of MAPK. The results of this study were consistent with those previously reported (Zheng et al., 2022). The expression levels of the phosphorylated PI3K and Akt proteins were higher in the 200 and 400 mg/L *Cyanotis arachnoidea* extract groups than in the insulin resistance group (*P* < 0.05). The glucose concentration was significantly lower in the cell culture medium than in the insulin resistance group. Thus, *Cyanotis arachnoidea* upregulates PI3K and Akt proteins, strengthens the signal transduction barrier of the PI3K/Akt signal pathway, inhibits the MAPK signal pathway, enhances glucose intake, improves insulin resistance, and play a hypoglycemic role in insulin-resistant pancreatic islet cells.

### Quantitative analysis

To ensure the efficacy and consistency, the content of quality markers should be monitored. UHPLC was uesd for the simultaneous quantification of the four quality marker compounds. The UHPLC analysis was quantitatively validated in terms of linearity, precision, repeatability, stability, and recovery.

According to method validation results of linearity, all calibration curves showed good linear ranges (*r*^2^ > 0.999) and simultaneously quantified the four quality markers in the samples (Table 4). Standard curves for the four components are shown in Fig. S1.

**Table 4:** Calibration curves for four potential active ingredient markers (*n* = 6).

Analyte	Calibration curve	*r* ^2^	Linear range (µg/mL)
20-Hydroxyecdysone	*Y* = 5.49 × 10^6^ *X* − 2.33 × 10^4^	0.9997	0.0344–0.8602
3-*O*-Acetyl-20-hydroxyecdysone	*Y* = 4.02 × 10^6^ *X* − 6.01 × 10^3^	1.0000	0.0092–0.2300
Ajugasterone C	*Y* = 4.38 × 10^6^ *X* − 3.91 × 10^3^	0.9996	0.0050–0.1256
2-*O*-Acetyl-20-hydroxyecdysone	*Y* = 4.61 × 10^6^ *X* − 4.35 × 10^3^	0.9998	0.0051–0.1281

The relative standard deviations (RSDs) for the precision, repeatability, and stability analyses of the four potential quality markers were all <3%. The result indicated that the instrument precision, method repeatability, and sample stability were good. Recovery of all four components was in the range of 98.72–101.44% which proved that the method was accurate (Table S5).

**Figure 8:**
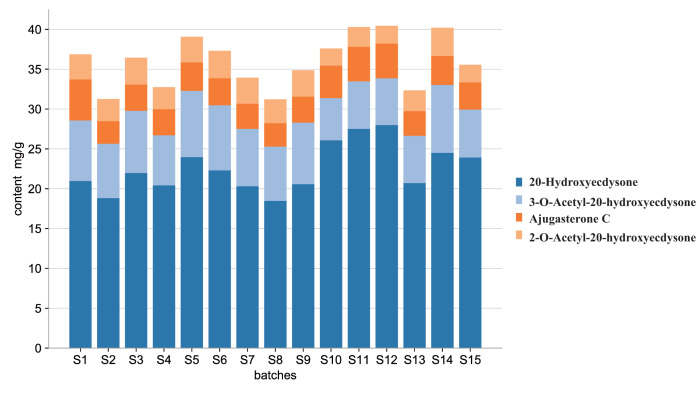
Histogram of quality markers content. Content of the four quality markers in 15 sample batches of *Cyanotis arachnoidea* collected from different origins. Different colors represent different components and 20-Hydroxyecdysone had the highest content of all four quality markers.

The contents of the four quality markers in fifteen batches were quantified, and the results were shown in Fig. 8. The most abundant component in *Cyanotis arachnoidea* was 20-Hydroxyecdysone (18.47–27.99 mg/g) followed by 3-*O*-Acetyl-20-hydroxyecdysone (5.30–8.54 mg/g), Ajugasterone C (2.84–5.16 mg/g) and 2-*O*-Acetyl-20-hydroxyecdysone (2.16–3.54 mg/g) (Table S6). The relative differences in the amounts of the components among the various batches indicate that the *Cyanotis arachnoidea* collected from different producing areas vary in composition and quality. The established method provided a reference for the control and evaluation of the quality of *Cyanotis arachnoidea*. The four components including 20-Hydroxyecdysone, 3-*O*-Acetyl-20-hydroxyecdysone, Ajugasterone C and 2-*O*-Acetyl-20-hydroxyecdysone could be considered as the quality markers of *Cyanotis arachnoidea*.

## Conclusion

In this study, we used chemical pattern recognition and network pharmacology to design and test a method of evaluating the overall quality of the TCM *Cyanotis arachnoidea*. We explored its quality markers and predicted the molecular mechanism in treating diabetes mellitus. UHPLC was used to characterize the chemical constituents of *Cyanotis arachnoidea* and analyze 15 sample batches collected from five different origins in Yunnan Province, China. The fingerprints of medicinal materials were established. The chromatographic peaks, HCA, PCA, and PLS-DA identified and screened 20-Hydroxyecdysone, 3-*O*-Acetyl-20-hydroxyecdysone, Ajugasterone C and 2-*O*-Acetyl-20-hydroxyecdysone as potential quality markers of *Cyanotis arachnoidea*. We predicted their biological activity *via* network pharmacology and molecular docking. We determined that *Cyanotis arachnoidea* may mediate its hypoglycemic efficacy by regulating its target genes encoding Akt1, TNF, IL-6, MAPK3, and JUN. The hypoglycemic property might also be explained by its impact on the PI3K/Akt, MAPK, TNF, TLR, and estrogen signaling, cancer, insulin resistance, and JAK-STAT pathways. *In vitro* experiments on pancreatic islet β-cells confirmed that *Cyanotis arachnoidea* enhances glucose uptake, improves insulin resistance and play a hypoglycemic role by promoting the PI3K/Akt and inhibiting the MAPK signal pathways. The foregoing results suggest that the quality markers in *Cyanotis arachnoidea* may act on the entire biological network rather than a single target gene. The present work demonstrated the integrity and complexity of *Cyanotis arachnoidea* function and also explained the mechanism of *Cyanotis arachnoidea* in treating diabetes mellitus with multiple components, gene targets, and signal pathways.

This study preliminarily identified the quality markers and potential pharmacological components of *Cyanotis arachnoidea*. Nevertheless, the hypoglycemic efficacy and modes of action of each active ingredient in this TCM must be explored and elucidated. This information will facilitate the utilization and optimization of *Cyanotis arachnoidea* for the clinical treatment of diabetes mellitus.

## Supplemental Information

10.7717/peerj.15948/supp-1 Supplemental Information 1 The standard curves of the four components.

10.7717/peerj.15948/supp-2 Supplemental Information 2 Supplemental Tables.

10.7717/peerj.15948/supp-3 Supplemental Information 3 Full-length uncropped blots.

10.7717/peerj.15948/supp-4 Supplemental Information 4 Raw data of relative protein expression.

## Additional Information and Declarations

### Data Availability

The following information was supplied regarding data availability: The raw measurements are available in the Supplemental File.
