# Supplementary figures and images for: Identification of quality markers for Cyanotis arachnoidea and analysis of its physiological mechanism based on chemical pattern recognition, network pharmacology, and experimental validation
Source: PeerJ. 2023 Sep 11;11:e15948. doi: 10.7717/peerj.15948 (PMC10501370; doi:10.7717/peerj.15948)

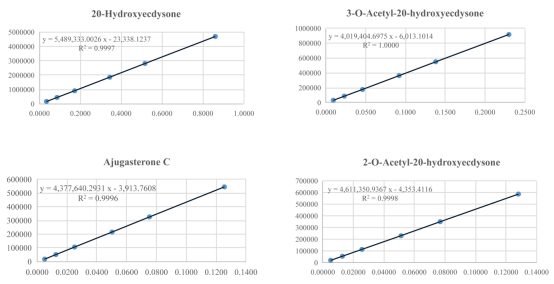

Supplement: Supplemental Information 1 [file peerj-11-15948-s001.png]

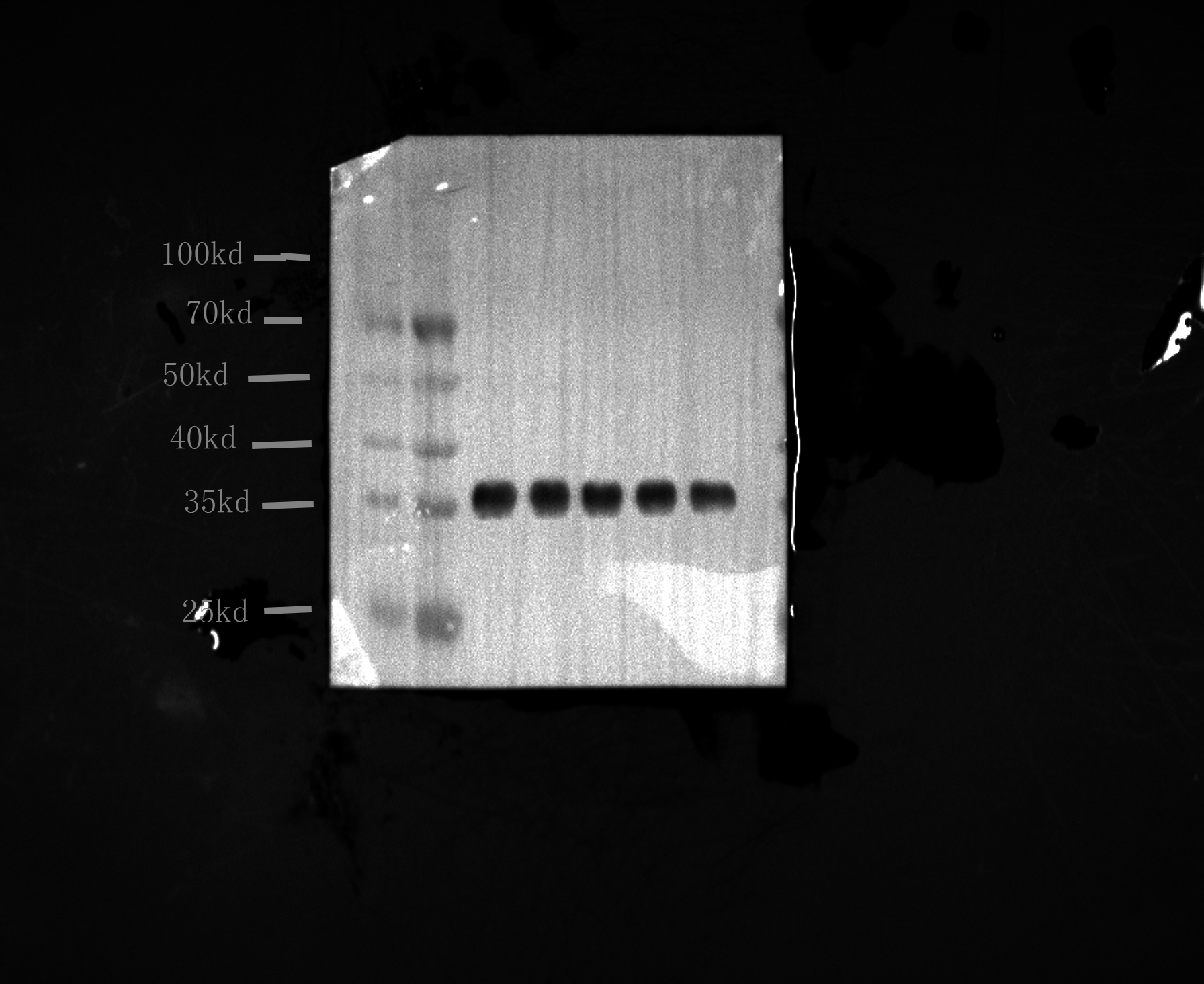

Supplement: Supplemental Information 3 [file peerj-11-15948-s003.zip › full-length uncropped blots/GAPDH.tif]

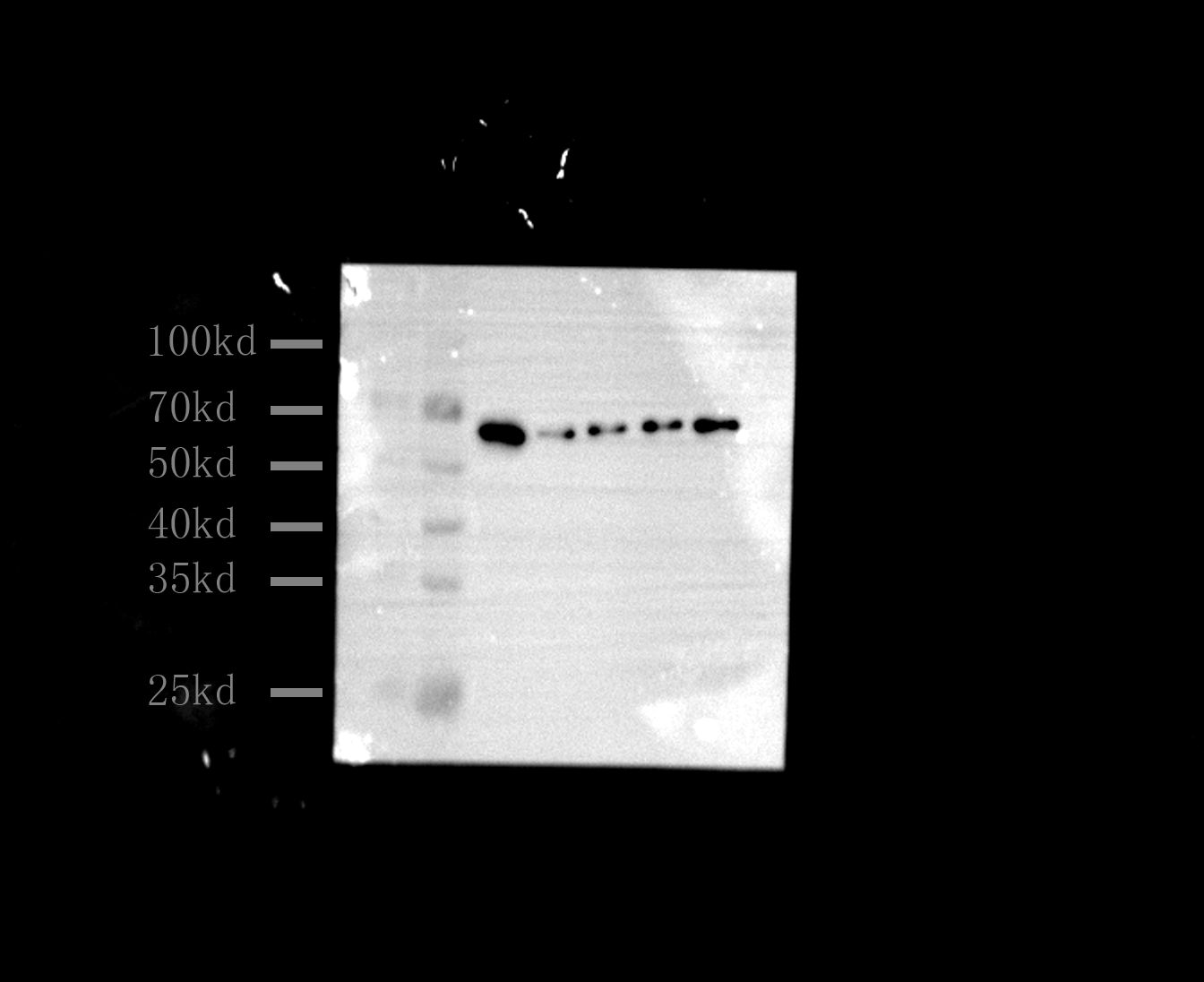

Supplement: Supplemental Information 3 [file peerj-11-15948-s003.zip › full-length uncropped blots/P-AKT.tif]

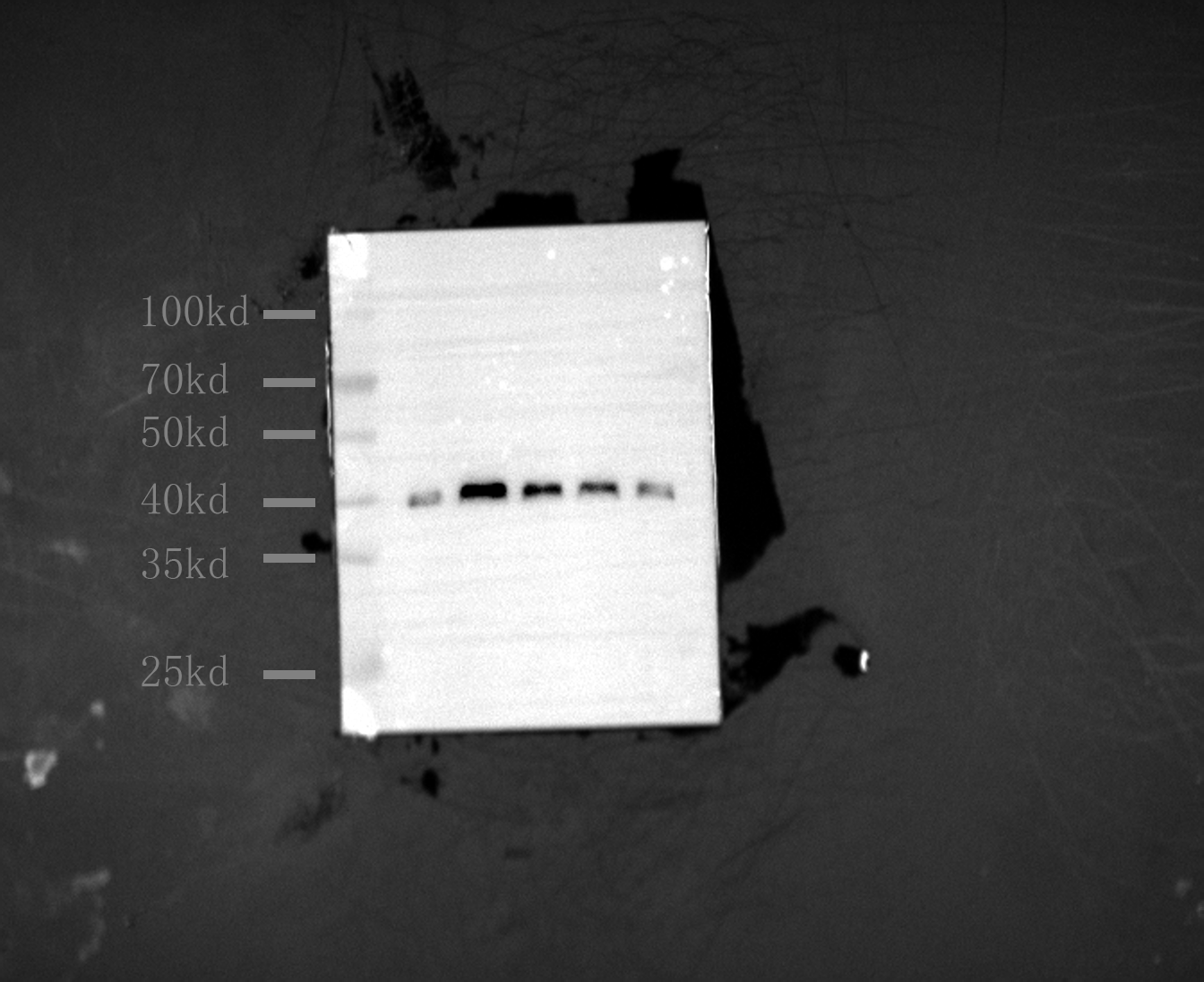

Supplement: Supplemental Information 3 [file peerj-11-15948-s003.zip › full-length uncropped blots/P-MAPK.tif]

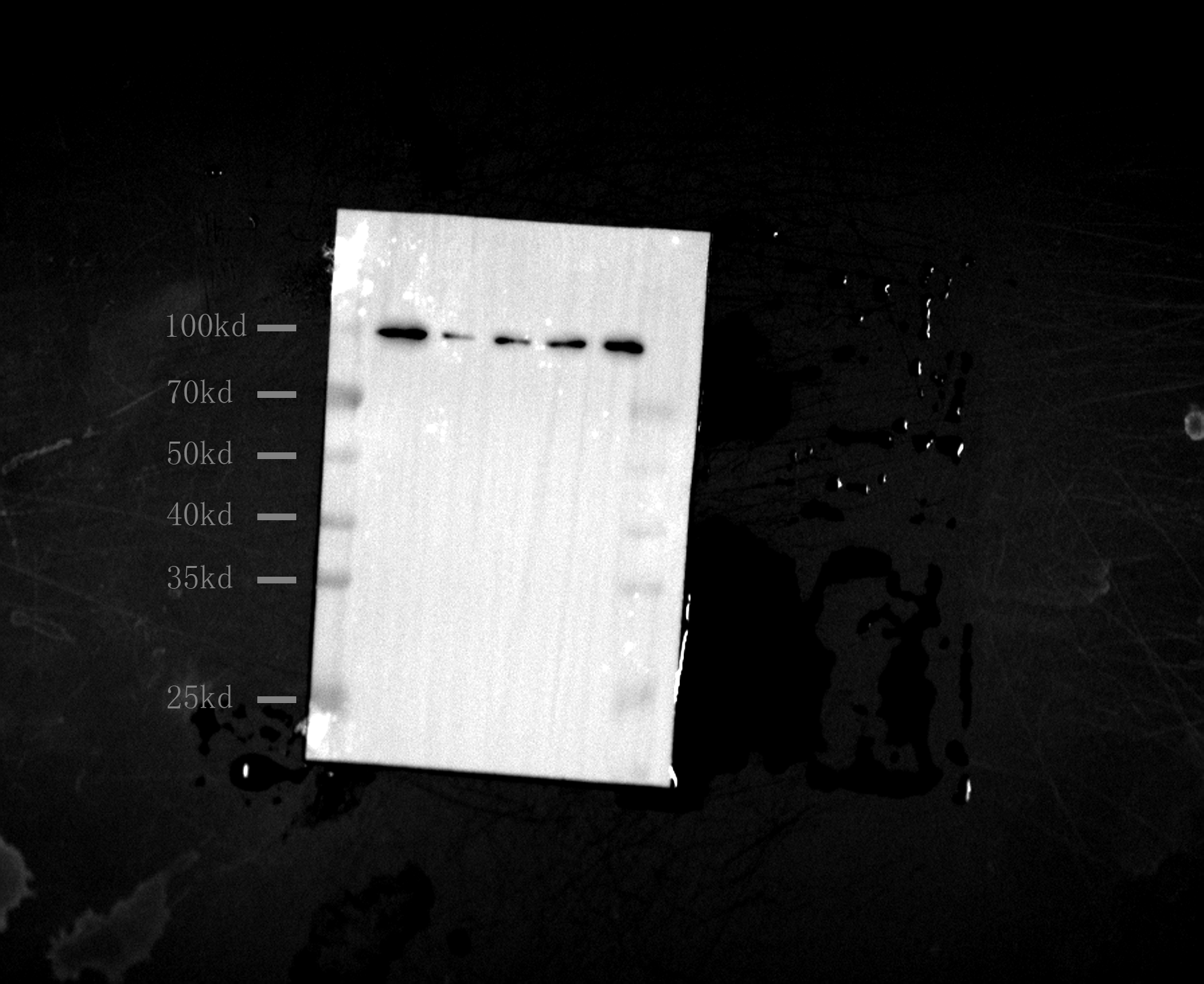

Supplement: Supplemental Information 3 [file peerj-11-15948-s003.zip › full-length uncropped blots/P-PI3K.tif]
