# Supplementary material for: Identification of quality markers for Cyanotis arachnoidea and analysis of its physiological mechanism based on chemical pattern recognition, network pharmacology, and experimental validation
Source: PeerJ. 2023 Sep 11;11:e15948. doi: 10.7717/peerj.15948 (PMC10501370; doi:10.7717/peerj.15948)
Supplement: Supplemental Information 2 [file peerj-11-15948-s002.doc]

Table S1 Origin and similarity of 15 batches samples of *Cyanotis arachnoidea*

| Batches | Batch number | Origins | Similarity Value |
| --- | --- | --- | --- |
| S1 | 2010161 | Anning | 0.998 |
| S2 | 2010162 | Anning | 0.999 |
| S3 | 2010163 | Anning | 0.999 |
| S4 | 2011254 | Menghai | 1.000 |
| S5 | 2011255 | Menghai | 0.999 |
| S6 | 2011256 | Menghai | 0.999 |
| S7 | 2010141 | Jinning | 0.999 |
| S8 | 2010142 | Jinning | 0.999 |
| S9 | 2010143 | Jinning | 0.998 |
| S10 | 2010164 | Jinping | 0.996 |
| S11 | 2010165 | Jinping | 0.997 |
| S12 | 2010166 | Jinping | 0.996 |
| S13 | 2011251 | Jinghong | 1.000 |
| S14 | 2011252 | Jinghong | 1.000 |
| S15 | 2011253 | Jinghong | 0.998 |

Table S2 Precision, repeatability, stability of UHPLC fingerprint of *Cyanotis arachnoidea* (RSD%)

| Peak NO. | Precision (n = 6) | | Reproducibility (n = 6) | | Stability (n = 6) | |
| --- | --- | --- | --- | --- | --- | --- |
|  | Relative retention time | Relative peak area | Relative retention time | Relative peak area | Relative retention time | Relative peak area |
| 1 | 0.08 | 1.04 | 0.08 | 1.68 | 0.12 | 0.5 |
| 2 | 0.08 | 1.23 | 0.08 | 2.64 | 0.11 | 0.83 |
| 3 | 0.09 | 1.27 | 0.07 | 2.42 | 0.13 | 2.93 |
| 4 | 0.15 | 2.83 | 0.1 | 1.79 | 0.21 | 2.09 |
| 5 | 0.1 | 0.5 | 0.06 | 0.55 | 0.19 | 0.44 |
| 6 | 0 | 0 | 0 | 0 | 0 | 0 |
| 7 | 0.03 | 1.99 | 0.03 | 2.09 | 0.04 | 2.75 |
| 8 | 0.09 | 2.62 | 0.05 | 2.58 | 0.09 | 2.81 |
| 9 | 0.12 | 0.06 | 0.07 | 0.19 | 0.13 | 0.25 |
| 10 | 0.12 | 2.3 | 0.07 | 2.55 | 0.13 | 2.1 |
| 11 | 0.12 | 1.86 | 0.07 | 1.46 | 0.13 | 1.08 |
| 12 | 0.12 | 0.89 | 0.07 | 1.36 | 0.14 | 1.24 |
| 13 | 0.13 | 0.27 | 0.08 | 1.71 | 0.15 | 1.28 |
| 14 | 0.14 | 2.34 | 0.1 | 2.39 | 0.18 | 2.86 |
| 15 | 0.19 | 2 | 0.13 | 2.44 | 0.27 | 2.93 |
| 16 | 0.2 | 2.33 | 0.13 | 1.84 | 0.28 | 1.39 |
| 17 | 0.19 | 0.53 | 0.13 | 2.41 | 0.28 | 0.62 |
| 18 | 0.19 | 2.31 | 0.14 | 2.33 | 0.27 | 1.56 |
| 19 | 0.2 | 1.53 | 0.13 | 2.81 | 0.28 | 2.69 |
| 20 | 0.19 | 2.89 | 0.14 | 2.77 | 0.28 | 2.96 |

Table S3 Glucose concentration in cell culture medium (n=3,  ± s)

| Group | Absorbance value | Glucose concentration ( mmol /L) |
| --- | --- | --- |
| Control | 0.43±0.01 | 5.55±0.16 |
| Model | 2.01±0.03 | 32.15±0.41^a^ |
| Low dose | 1.01±0.04 | 16.06±0.58^b^ |
| Medium dose | 0.93±0.03 | 13.97±0.041^b^ |
| High dose | 0.88±0.03 | 13.19±0.043^b^ |

a: Compared with the blank control group, *P*＜0.05

b: Compared with the model group, *P*＜0.05

Table S4 The relative protein expression of phosphorylated PI3K, AKT and MAPK

|  | Control | Model | Low dose | Medium dose | High dose |
| --- | --- | --- | --- | --- | --- |
| P-PI3K 1/GAPDH | 0.816819317 | 0.149513776 | 0.269607843 | 0.486650485 | 0.65776699 |
| P-PI3K 2/GAPDH | 0.902997502 | 0.141004862 | 0.31004902 | 0.447815534 | 0.609223301 |
| P-PI3K 3/GAPDH | 1.14029975 | 0.151944895 | 0.370098039 | 0.669902913 | 0.910194175 |
| P-AKT 1/GAPDH | 1.152789342 | 0.18719611 | 0.24877451 | 0.381067961 | 0.564320388 |
| P-AKT 2/GAPDH | 1.024146545 | 0.205429498 | 0.200980392 | 0.390776699 | 0.569174757 |
| P-AKT 3/GAPDH | 0.835553705 | 0.15802269 | 0.305147059 | 0.519417476 | 0.730582524 |
| P-MAPK 1/GAPDH | 0.144879267 | 0.730551053 | 0.550245098 | 0.366504854 | 0.27184466 |
| P-MAPK 2/GAPDH | 0.143630308 | 0.713533225 | 0.515931373 | 0.383495146 | 0.280339806 |
| P-MAPK 3/GAPDH | 0.167360533 | 0.852106969 | 0.640931373 | 0.535194175 | 0.372572816 |

Table S5 Precision, repeatability, stability and recovery results of quantitative analysis of the four potential active ingredients in *Cyanotis arachnoidea*

| **Analyte** | **Precision**  **(n = 6)** | **Reproducibility (n = 9)** | **Stability**  **(n = 6)** | **Recovery**  **(*n***= **9)** | |
| --- | --- | --- | --- | --- | --- |
|  | RSD (%) | RSD (%) | RSD (%) | Mean (%) | RSD (%) |
| 20-Hydroxyecdysone | 0.18 | 1.2 | 0.45 | 101.44 | 1.06 |
| 3-O-Acetyl-20-hydroxyecdysone | 0.51 | 0.94 | 0.61 | 99.9 | 2.47 |
| Ajugasterone C | 0.25 | 1.18 | 1.6 | 99.58 | 2.08 |
| 2-O-Acetyl-20-hydroxyecdysone | 0.35 | 1.75 | 1.38 | 98.72 | 2.12 |

Table S6 The contents of the quality markers in fifteen batches of *Cyanotis arachnoidea* (mg/g)

| Batches | 20-Hydroxyecdysone | 3-O-Acetyl-20-hydroxyecdysone | Ajugasterone C | 2-O-Acetyl-20-hydroxyecdysone |
| --- | --- | --- | --- | --- |
| S1 | 20.97 | 7.60 | 5.16 | 3.13 |
| S2 | 18.83 | 6.82 | 2.84 | 2.77 |
| S3 | 21.97 | 7.81 | 3.29 | 3.36 |
| S4 | 20.42 | 6.28 | 3.29 | 2.75 |
| S5 | 23.96 | 8.33 | 3.57 | 3.21 |
| S6 | 22.30 | 8.18 | 3.39 | 3.44 |
| S7 | 20.31 | 7.21 | 3.16 | 3.26 |
| S8 | 18.47 | 6.83 | 2.93 | 3.00 |
| S9 | 20.58 | 7.72 | 3.26 | 3.32 |
| S10 | 26.08 | 5.30 | 4.06 | 2.16 |
| S11 | 27.51 | 5.97 | 4.33 | 2.47 |
| S12 | 27.99 | 5.87 | 4.35 | 2.25 |
| S13 | 20.72 | 5.92 | 3.10 | 2.60 |
| S14 | 24.50 | 8.54 | 3.62 | 3.54 |
| S15 | 23.91 | 6.03 | 3.40 | 2.23 |
